# Neck abscess due to *Salmonella* Choleraesuis: case study and literature review

**DOI:** 10.1099/jmmcr.0.005109

**Published:** 2017-09-01

**Authors:** Ryoji Sugimoto, Hirotaka Suzuki, Takahito Nei, Ayaka Tashiro, Yohei Washio, Kazunari Sonobe, Yuzo Nakamura, Nozomu Wakayama, Shunta Inai, Hidemasa Izumiya

**Affiliations:** ^1^​ Department of Clinical Laboratory, Nippon Medical School Hospital, Japan; ^2^​ Department of Infection Control and Prevention, Nippon Medical School Hospital, Japan; ^3^​ Department of Oto-Rhino-Laryngology and Head and Neck Surgery, Nippon Medical School Hospital, Japan; ^4^​ Department of Bacteriology I, National Institute of Infectious Diseases, Japan

**Keywords:** neck abscess, patient without HIV infection, *Salmonella* Choleraesuis, urgent incision, nalidixic acid resistant

## Abstract

**Introduction.** We herein describe a case with a neck abscess due to non-typhoidal *Salmonella* (NTS). NTS habitually reside in our environment and colonize all animals including mammals. Colonizations of pigs, chickens, cows and sheep are important because food poisoning episodes in human are often associated with meat. Extra-intestinal infection due to NTS has numerous presentations and complications, with aortic aneurysms being common.

**Case presentation.** A 26-year-old Japanese male complaining of left-sided neck swelling was referred to our hospital for a suspected deep neck abscess. An enhanced computed tomography scan of the neck revealed a low density lesion in the left-sided deep neck area, and consequently the patient underwent urgent incision and drainage. After this urgent operation, *Salmonella* Choleraesuis was isolated from a greyish-white abscess. The patient ultimately recovered with antimicrobial administration, though re-incision for lymphadenectomy was necessary. The neck abscess may have developed because he had eaten raw meat. Furthermore, untreated diabetes mellitus was diagnosed at presentation.

**Conclusion.** *Salmonella enterica* serovar Choleraesuis infections are rare in Japan. NTS are generally recognized as important pathogens in food poisoning globally, and attention is required to avoid the development of extra-intestinal infections. In Japan, the increasing lifestyle diversity in recent years highlights the importance of recognizing rare infections.

## Abbreviations

CT, computed tomography; IGRA, interferon-gamma releasing assay; MLST, multilocus sequence typing; NTS, non-typhoidal Salmonella.

## Introduction

The genus *Salmonella* comprises more than 2500 serotypes, and non-typhoidal *Salmonella* (NTS), excluding *Salmonella* Typhi and *Salmonella* Paratyphi A causing enteric fever, are generally recognized as a cause of food poisoning globally. NTS habitually reside in our environment, and colonize all animals, birds, reptiles, and mammals. Colonizations of pigs, chickens, cows and sheep are especially important because food poisoning episodes are often associated with meat. Extra-intestinal infection due to NTS has numerous presentations and complications, with aortic aneurysms being common. *Salmonella* Choleraesuis can adapt to a porcine host and is recognized as an important pathogen causing a cholera-like illness. It also has high pathogenicity in humans, and the capacity to cause extra-intestinal infections. Herein, we describe a case of left-sided deep neck abscess due to *S*. Choleraesuis.

## Case report

A 26-year-old apparently healthy Japanese male visited a neighbourhood clinic for left-sided neck swelling which had persisted for the previous two weeks. Computed tomography (CT) scanning showed neck lymphadenitis. Furthermore, the patient was also diagnosed with untreated diabetes mellitus based on his haemoglobin A1c and occasional plasma glucose exceeding 14.8 % and 522 mg dl^−1^, respectively. After hospitalization, antibiotics were administered for the neck lymphadenitis alongside therapy for diabetes mellitus including insulin injections. The patient was initially given ceftriaxone (2 g every 12 h) but treatment was switched to tazobactam-piperacillin (4.5 g every 8 h) and ciprofloxacin (300 mg every 12 h) based on lack of changes in his serum inflammatory reaction parameters. However, despite the change in antimicrobials, the condition showed no remission and the patient was referred to the department of Oto-Rhino-Laryngology and Head and Neck Surgery of our hospital. His past and familial medical histories were unremarkable. He was a current smoker (nine packs per year) and occasional drinker. He had never been to a foreign country and had no animal contact. He was not highly active sexually and was heterosexual. However, he often ate raw meat.

**Fig. 1.**
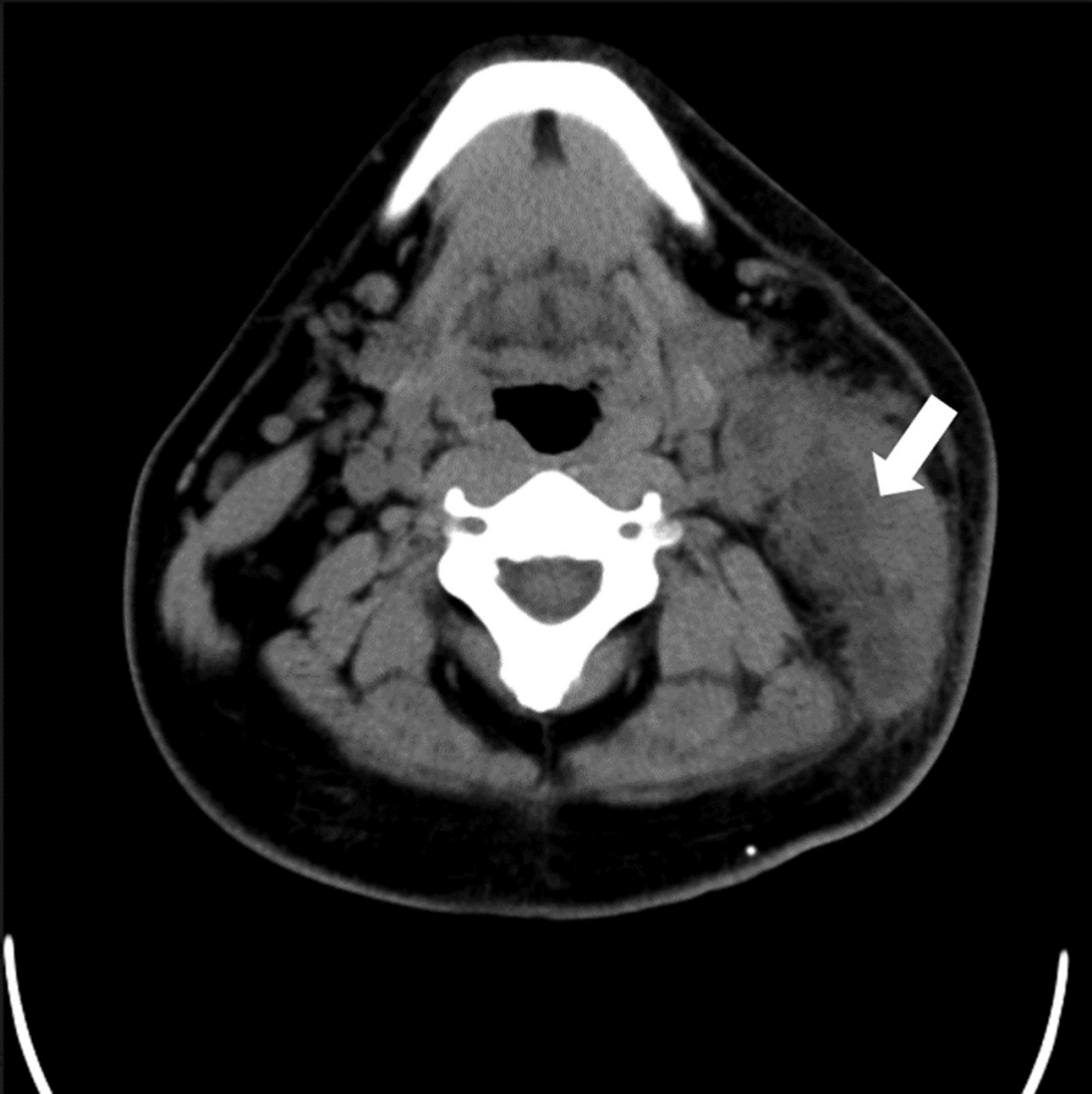
Neck CT scans on admission. Low density masses are recognized (white arrow) in the left deep neck region.

A neck CT scan obtained at the former facility revealed a low density area in the deep left-sided area, suggesting an abscess (Fig. 1). Blood examination data at the first visit to our hospital are shown in Table 1. The patient was admitted on that day and underwent urgent incision and drainage of the abscess. Post-operatively, his status was good with antimicrobial administration (tazobactam-piperacillin, 4.5 g every 8 h) and daily irrigation of the wounded area. Subsequently, a species of *Salmonella* was isolated from the abscess and the serotype was identified as 6,7 : c : 1.5, raising suspicion of *S*. Choleraesuis. We planned to continue antimicrobial administration, however, the patient left the hospital against medical advice.

**Table 1.** Laboratory data of the patient with neck abscess on admission to Nippon Medical School Hospital

**Parameter**	**Amount detected**
Blood cell counts	
White blood cells	13 100 µl^−1^
Neutrophils	70.6 %
Lymphocytes	22.0 %
Eosinophils	1.9 %
Red blood cells	501×10^4^ µl^−1^
Haemoglobin	14.6 g dl^−1^
Platelets	43.0×10^4^ µl^−1^
Biochemical measurements	
Aspartate aminotransaminase	19 IU l^−1^
Alanine aminotransferase	23 IU l^−1^
Lactose dehydrogenase	169 IU l^−1^
γ-Glutamyl transferase	90 IU l^−1^
Sodium	136 mEq l^−1^
Potassium	4.2 mEq l^−1^
Chloride	101 mEq l^−1^
Blood urea nitrogen	10.8 mg dl^−1^
Creatinine	0.72 mg dl^−1^
Total protein	7.3 g dl^−1^
Albumin	3.8 g dl^−1^
Plasma glucose	88 mg dl^−1^
Haemoglobin A1c	13.80 %
Serum inflammatory marker	
C reactive protein	3.03 mg dl^−1^

At the first visit after leaving our hospital, the patient complained of recurrent swelling of the left neck and was re-admitted for recurrence of neck lymphadenitis. He was administered levofloxacin (500 mg every 24 h), but the treatment was switched to ceftriaxone (2 g every 12 h) based on resistance to nalidixic acid. A follow-up neck CT showed a residual lesion with lymphadenitis, and the patient underwent re-incision and lymphadenectomy on the 21st hospital day. The extracted tissue, including a lymph node, was positive by acid-fast staining. However, no acid-fast bacilli grew, despite culture for 6 weeks in liquid medium. On the other hand, tissue pathological findings revealed granuloma formation. We initially suspected accompanying lymphadenitis tuberculosa, but there was no evidence of *Mycobacterium tuberculosis* complex related genes by PCR. We observed what appeared to be the onset of tuberculosis, but the patient recovered with ceftriaxone administration only for salmonellosis. He was discharged on the 34th hospital day. Approximately two years after the discharge, there has been no recurrence of neck swelling.

**Fig. 2.**
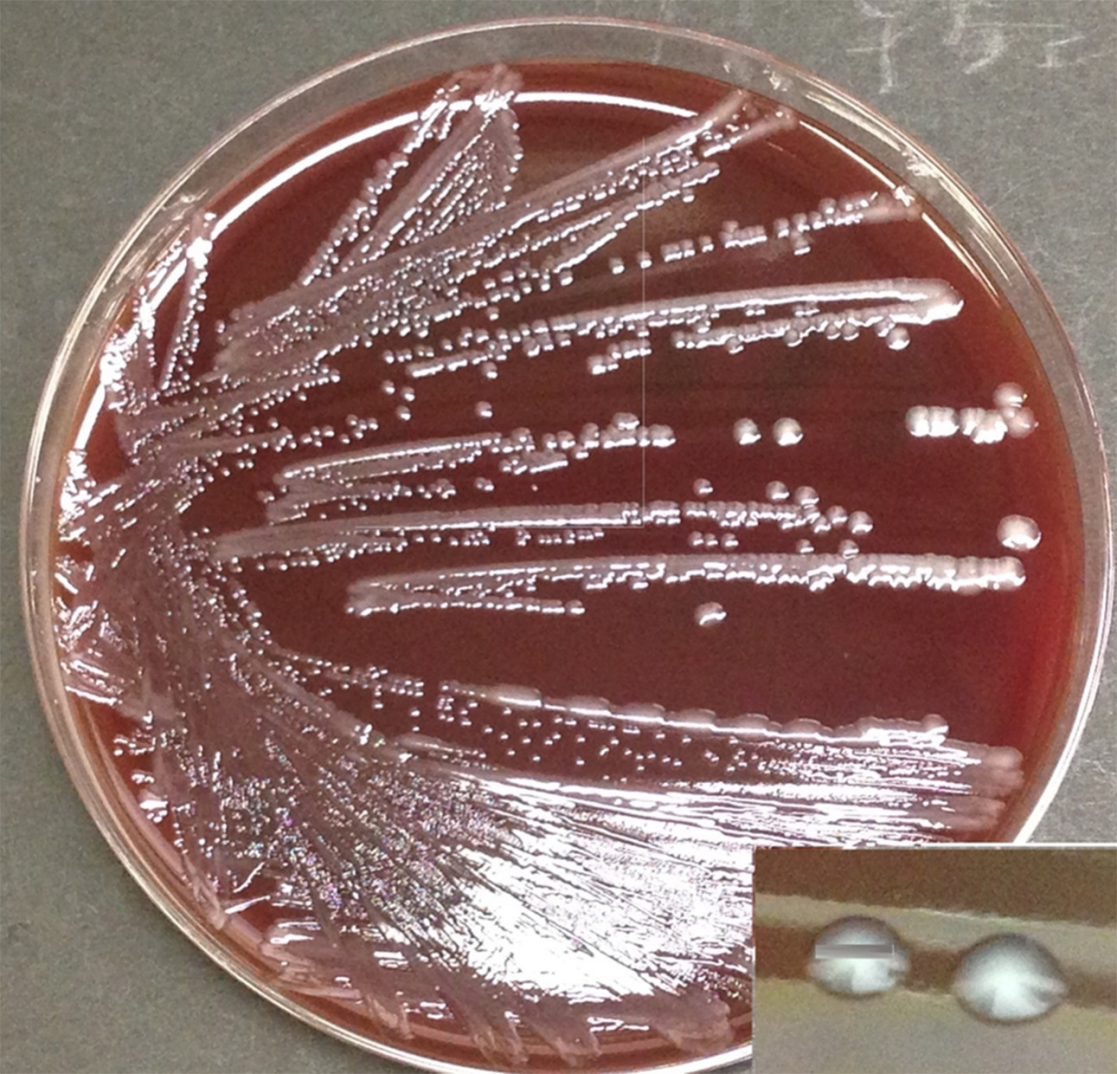
Isolation of colonies from cultured neck abscess samples on the agar medium, and high-power view of colonies (lower right panel). Colonies grew rapidly, and white transparent colonies were observable at 24 h.

Smears of the neck abscess specimens showed Gram-negative bacilli with uneven body lengths. Aerobic culture on 5 % sheep serum agar (Eiken) and MacConkey agar (Oriental Yeast Co.) at 35 °C, revealed a clear whitish colony on both media within 24 h of starting the cultures (Fig. 2). We identified the isolate as *S*. Choleraesuis using WalkAway with a MicroScan series NENC1J panel. On the other hand, serological typing using a commercial detection kit (Denka Seiken) yielded 6,7 : c : 1.5. The isolate showed no hydrogen sulfide production, and we thus considered the isolate to be *S*. Choleraesuis *sensu stricto* or *Salmonella* Typhisuis [1]. Fermentations of d-tartrate and sorbitol were confirmed by 48 h observation but did not manifest within 24 h [2]. Furthermore, identification by the multilocus sequence typing (MLST) method revealed no homology with any of the serotypes in the database. However, though an allele type of *sucA* was confirmed to be type 393, ST145 was the type nearest that of the isolate other than the *sucA* type [3]. In serotype group 6,7 : c : 1.5, ST145 showed full matching with *S*. Choleraesuis var. Kunzendorf. However, some strains cannot be classified into *S*. Choleraesuis var. Kunzendorf based on hydrogen sulfide production and MLST identification [4]. Moreover, previous reports showed that ST145 includes some strains of *S*. Chorelaesuis *sensu stricto* [1, 5]. Thus, we decided that the isolate must be *Salmonella enterica* subsp. *enterica* serovar Choleraesuis *sensu stricto* based on the lack of hydrogen sulfide production and the absence of d-tartrate and sorbitol fermentations. Antimicrobial susceptibilities except to nalidixic acid were confirmed using a MicroScan series NENC1J panel based on the Clinical and Laboratory Standards Institute guidelines (M100-S22). Resistance to nalidixic acid was confirmed using the disc diffusion method, and the results are shown in Table 2 along with isolate characteristics.

**Table 2.** Characteristics and antibiotic susceptibilities of the *Salmonella* Choleraesuis *sensu stricto* isolates

**Characteristic tested**	**Result**
Serotyping	6, 7 : c : 1.5
Anti-O antigen	Antisera group 7
Hydrogen sulfide production	−
Resolution capacities	
Lysine	+
d-Tartrate	+*
Sorbitol	+*
**Multilocus sequence typing**	**Allele type**
*aroC*	36
*dnaN*	31
*hemD*	35
*hisD*	14
*purE*	26
*sucA*	393
*thrA*	8
Serotyping	na
**Antibiotic susceptibilities**	**MIC (μg ml^−1^)**
Ampicillin	>8
Sulbactum/ampicillin	16
Piperacillin	>64
Tazobactum/piperacillin	<16
Cefotaxim	<1
Ceftadizim	<4
Cefepime	<2
Imipenem	<1
Meropenem	<1
Aztreonam	<4
Amikacin	<4
Gentamicin	<2
Minocycline	>8
Levofloxacin	2
Nalidixic acid	Resistant†
Trimethoprim/sulfamethoxazole	<2
Fosfomycin	<4

*Judged by 48 h incubation.

†Judged by disc diffusion method.

+, Positive; −, negative; na, not applicable.

## Discussion

NTS are now regarded as one of the pathogens commonly causing bloodstream infections globally. Human immunodeficiency virus carriers, as well as neonates and young children, are considered to be at especially high risk for NTS bloodstream infections, and vulnerable patients are increasing in number worldwide [6, 7]. Recently, 3.4 million patients were estimated to have invasive NTS infections annually in the world, with the incidence being five times higher in Africa than in other areas [6]. Furthermore, emerging antimicrobial resistance of NTS is developing in Kenya and reinforcement of anti-infection measures is now regarded as urgent [8]. Indeed, acquisition of multidrug resistance is also an important global issue [9].

The most common route of NTS infection in humans is food poisoning. However, we should keep in mind the development of extra-intestinal infections in immunocompromised patients [10, 11]. With these extra-intestinal NTS infections, there is a high risk of an infected aneurysm occurring in large arteries [12], but a previous report found that 20 % of extra-intestinal NTS infections developed into abscesses [13]. Thus, abscess formation is not rare with extra-intestinal NTS infections. Furthermore, 74 % of extra-intestinal NTS infections are reportedly due mainly to eight *Salmonella* serotypes, including *S*. Choleraesuis [11]. Within the *S*. Choleraesuis family, there are no special features or characteristic symptoms of infectious disease in humans. Thus, gastrointestinal symptoms are most common [12], similar to other NTS infections, and can lead to extra-intestinal infections including abscess formation [14–17]. Despite extra-intestinal infection development, we detected no symptoms suggesting gastrointestinal infections in our reported patient [14, 15, 18]. Furthermore, considering only neck abscess due to *Salmonella* species, there are several reports of cases with concomitant diabetes mellitus [14, 17, 18] or liver cirrhosis [15]. Interestingly, the development of anti-interferon-gamma autoantibodies in a healthy individual can lead to opportunistic infections including invasive NTS infections [19, 20]. Thus, extra-intestinal NTS infection, rather than an immunocompromised state, is considered to be the main background factor contributing to the development of invasive disease.

The reason for invasive NTS infection developing in this case is unknown, but the patient's intake of raw meat might have been a factor. Interestingly, previous reports have described infections with different serotypes of NTS [12]. For example, previously, *S*. Choleraesuis was more often isolated as a pathogen of invasive NTS infection in Taiwan than in the USA [12], but recent reports indicate that this difference in detection rates is decreasing [21]. Furthermore, in Japan, *S*. Choleraesuis *sensu stricto* is often isolated from pigs in eastern areas including Tokyo, while *S*. Choleraesuis var. Kunzendorf tends to be found in western areas including Kyushu. This phenomenon may shed light on the origins of these organisms [22]. In other words, species distribution is greatly influenced by lifestyle and environmental factors, possibly clarifying why *S*. Choleraesuis *sensu stricto* was identified in our case. However, detailed information about the patient's habit of eating raw meat is lacking. He recalled possibly having eaten pork, but provided no other details. We do not know why *S.* Choleraesuis caused only a neck lesion. Though the patient had some dental caries, we identified neither oral damage nor small abscesses in his oral cavity. Moreover, as mentioned above, invasive NTS infection is closely related to a rise in the endogenous anti-interferon-gamma autoantibody level. However, our patient’s sera were not assayed for this autoantibody. Based on testing with the interferon-gamma releasing assay (IGRA), without positive/negative control failure, he did not have anti-interferon-gamma autoantibody [19].

A relationship between extra-intestinal NTS infection and granuloma formation has been reported [23–26]. Though we recognized granuloma in an extracted specimen and the acid-fast staining result was positive, we continued antimicrobial administration for salmonellosis. The PCR assay, acid-fast bacilli culture and IGRA yielded no positive results. Even after the second discharge, we continued careful observation. Neither the abscess nor neck lesions recurred. We ultimately decided that the lymphadenitis was not due to tuberculosis.

We assessed the reliability of the isolation data by confirming the identity of the isolate. At most health facilities, microbiological tests depend on automatic analyser use, but erroneous identifications are common. It is important to detect the fermentations of d-tartrate and sorbitol for differentiating *Salmonella enterica* serotype 6,7 : c : 1.5, according to some laboratory manuals. However, despite repeated tests for these fermentations, only negative results were obtained. We advocate performing these tests after more than 48 h of culture, not 24 h. Indeed, it is difficult to perform MLST analysis for all isolated NTS, though it is possible to achieve identification based on serotyping and microbiological characteristics. Hence, it is important to combine approaches when identifying species of the genus *Salmonella*, relying on both test results and clinical experience.

In conclusion, we have described the course of a presentation with a deep neck abscess due to NTS, a rather rare infection in Japan. The reason for NTS causing a deep neck abscess was not clarified, but dietary intake of raw meat was considered to be a factor. Extra-intestinal NTS infections are common worldwide but not in developed countries like Japan. However, invasive NTS infections may increase due to changes in lifestyle and other habits. Thus, our present case highlights the importance of considering zoonoses in the differential diagnosis. NTS have been recognized as important pathogens in food-poisoning globally, and attention has been required to avoid the development of extra-intestinal infections. The increasing lifestyle diversity in recent years also highlights the importance of recognizing NTS infections.
